# Preserving Retinal Structure and Function with the Novel Nitroxide Antioxidant, DCTEIO

**DOI:** 10.1007/s11064-023-03978-w

**Published:** 2023-07-14

**Authors:** Cassie L. Rayner, Steven E. Bottle, Alexander P. Martyn, Nigel L. Barnett

**Affiliations:** 1 Clem Jones Centre for Regenerative Medicine, Faculty of Health Sciences and Medicine, Bond University, 14 University Drive, Robina, Gold Coast, QLD 4226 Australia; 2 Queensland Eye Institute, South Brisbane, QLD 4101 Australia; 3 School of Physical and Chemical Sciences, Queensland University of Technology, Brisbane, QLD 4000 Australia; 4 Cancer and Ageing Research Program (CARP), Princess Alexandra Hospital, Brisbane, QLD 4102 Australia

**Keywords:** Oxidative stress, Ischemia, Neuroprotection, Retina, Nitroxide, Antioxidant

## Abstract

Oxidative stress is a major contributor to progressive neurodegenerative disease and may be a key target for the development of novel preventative and therapeutic strategies. Nitroxides have been successfully utilised to study changes in redox status (biological probes) and modulate radical-induced oxidative stress. This study investigates the efficacy of DCTEIO (5,6-dicarboxy-1,1,3,3-tetraethyllisoindolin-2-yloxyl), a stable, kinetically-persistent, nitroxide-based antioxidant, as a retinal neuroprotectant. The preservation of retinal function following an acute ischaemic/reperfusion (I/R) insult in the presence of DCTEIO was quantified by electroretinography (ERG). Inflammatory responses in retinal glia were analysed by GFAP and IBA-1 immunohistochemistry, and retinal integrity assessed by histology. A nitroxide probe combined with flow cytometry provided a rapid technique to assess oxidative stress and the mitigation offered by antioxidant compounds in cultured 661W photoreceptor cells. DCTEIO protected the retina from I/R-induced damage, maintaining retinal function. Histological analysis showed preservation of retinal integrity with reduced disruption and disorganisation of the inner and outer nuclear layers. I/R injury upregulated GFAP expression, indicative of retinal stress, which was significantly blunted by DCTEIO. The number of ‘activated’ microglia, particularly in the outer retina, in response to cellular stress was also significantly reduced by DCTEIO, potentially suggesting reduced inflammasome activation and cell death. DCTEIO mitigated oxidative stress in 661W retinal cell cultures, in a dose-dependent fashion. Together these findings demonstrate the potential of DCTEIO as a neuroprotective therapeutic for degenerative diseases of the CNS that involve an ROS-mediated component, including those of the retina e.g. age-related macular degeneration and glaucoma.

## Introduction

Almost all retinal diseases are progressive and lead to vision loss and eventual blindness [1]. Slowing or avoiding neuronal death is the most effective way to treat retinal disease, with the identification and application of neuroprotective agents becoming a focal point of modern medicine [2]. Although the exact etiology of neurodegenerative diseases, including glaucoma, age-related macular degeneration (AMD) and diabetic retinopathy is yet to be elucidated, changes to the redox status through the overproduction of reactive oxygen species (ROS) and failure of cellular antioxidant defence mechanisms i.e. oxidative stress, is an established contributing factor. Cumulative oxidative stress induces cellular damage, impairment of the deoxyribonucleic acid (DNA) repair system and mitochondrial dysfunction, so accelerating the ageing process and the development of neurological disorders. Only recently has neuroinflammation and immune response been determined to be part of the sequence of pathological events leading to optic neuropathy, with emerging evidence supporting their coexistence in numerous central and peripheral pathologies [3].

The redox system plays a key role in cell homeostasis and survival by modulating cell signalling, defence and detoxification [4]. ROS are natural by-products of aerobic respiration by the mitochondria and, at low to moderate levels, are crucial to the initiation and dysregulation of immune activity developing the inflammatory response [3]. Inflammation is an integral part of the body’s physiological repair mechanism fighting off a multitude of threating conditions [5], however like oxidative stress, if it remains unresolved has the capacity to propagate neuronal injury and become pathological. While not the primary cause of destruction in these diseases, secondary damage mediated by the inflammatory response constantly disrupts the return to homeostasis [5], resulting in the eventual loss of the retinal cells and consequent blindness.

Nitroxide-based drugs (e.g. TEMPOL) have proven effective in modulating radical-induced oxidative stress and inflammation, and preventing or treating vascular, ocular and other pathological and age-related diseases, by modulating antioxidant enzymes and genes that control distinct immune and anti-inflammatory responses [6]. Nitroxides deliver potent antioxidant action and attenuate ROS in various models of oxidative stress ascribed to their superoxide dismutase (SOD) or redox and radical-scavenging actions [7–12]. Nitroxides have an additional advantage over other antioxidants in that the effective concentration can be relatively constant during the antioxidant reaction, because the nitroxide acts as a catalyst [13]. Their high cell permeability also makes them ideal therapeutics as they can access various cellular compartments and directly target the major site and organelle of cellular radical production, the mitochondria [14–16]. This unique cellular targeting ability also renders nitroxides highly suitable as in vivo biological probes for the detection of oxidative stress.

The unique ability of a profluorescent, reversibly responsive, nitroxide-based probe, methyl ester tetraethylrhodamine nitroxide (ME-TRN), to detect oxidative stress production in vivo and in real-time has been demonstrated [17]. Furthermore, it has been used to quantify the protection offered by the novel, nitroxide-based parent antioxidant, 5,6-dicarboxy-1,1,3,3-ttetraethyllisoindolin-2-yloxyl (DCTEIO) [18]. These studies demonstrated that DCTEIO is capable of not only ameliorating the production of ROS during an ischaemic insult when administered before ischaemic/reperfusion (I/R) injury, but also possesses the ability to reverse the accumulation of ROS when administered after an ischaemic insult during reperfusion. These data were however based on ‘short term’ studies, and further investigations into ‘long term’ effects were warranted. Here the potential ‘long term’ protection (8 days) offered by DCTEIO is investigated by combining the pre- and post-ischaemic administration of antioxidant. A rapid technique for quantifying oxidative stress in the 661W retinal photoreceptor cell line [19], was also established by combining the use of the ME-TRN probe with flow cytometry. The response of the ME-TRN probe to changes in the cellular redox environment when subjected to oxidative stress and antioxidant intervention is reported.

## Materials and Methods

### Animals and Treatments

Albino Sprague–Dawley rats (female, ~ 250 g) obtained from the Animal Resources Centre (Canning Vale, WA, Australia) at 8 weeks of age, were housed at Herston Medical Research Centre Animal Facility (Royal Brisbane & Women’s Hospital, Australia). Animals were maintained in temperature and humidity-controlled rooms (~ 37 °C and 60–70% respectively), with food and water available ad libitum. A 12:12 h light/dark cycle (lights on at 7 a.m.) was used, with illumination provided by overhead fluorescent white lights.

**Fig. 1:**
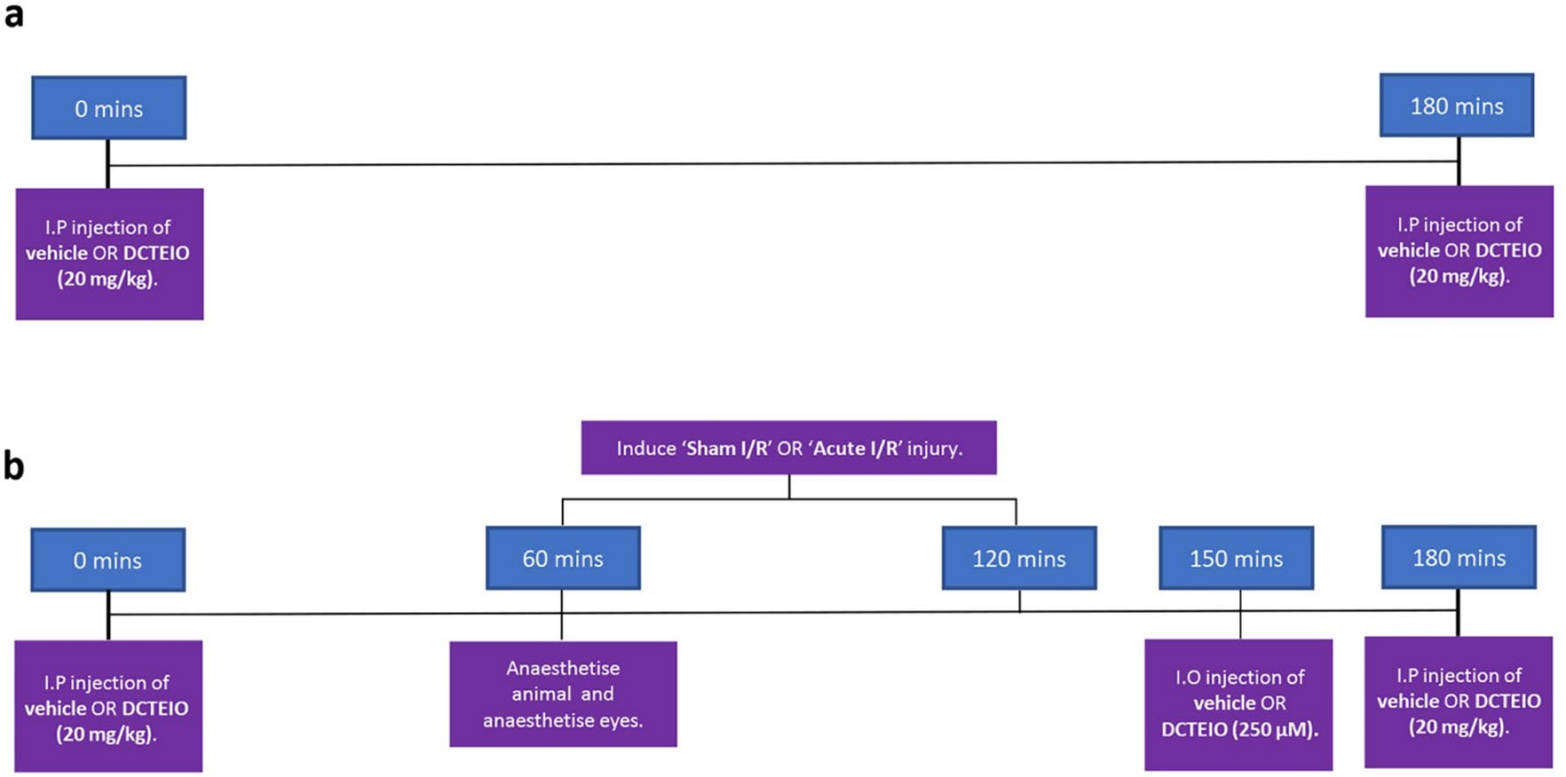
Treatment timelines for **a** I.P control group and **b** sham I/R and acute I/R treatment groups

Animals were utilised to study the potential ‘long term’ neuroprotective effect of the novel nitroxide-based antioxidant, DCTEIO. Rats were administered an intraperitoneal (I.P) injection of either vehicle or DCTEIO (20 mg/kg), one hour prior to inducing ‘sham I/R’ or ‘acute I/R’ injury. Intraocular (I.O) injections of vehicle or DCTEIO (250 µM) were administered to both eyes 30 min into the reperfusion phase, followed by a second I.P. injection 60 min post I/R or sham treatment (see Fig. 1). The generation of ROS following I/R injury provides a known in vivo pro-oxidant condition [20], upon which we based and quantified the extent of neuroprotection offered by antioxidant intervention. ‘I.P injection only’ animals were also included to discern any potential systemic drug effects (Fig. 1).

Animals were divided into six treatment groups for neuroprotection studies: (i) I.P: vehicle, (ii) sham I/R injury: vehicle, (iii) acute I/R injury: vehicle, (iv) I.P: DCTEIO, (v) sham I/R injury: DCTEIO and (vi) acute I/R injury: DCTEIO.

Experiments were conducted in accordance with the ‘Animal Care and Protection Act (QLD) 2001’, ‘The Australian Code of Practice for the Care and Use of Animals for Scientific Purposes’ and the ‘ARVO statement for the Use of Animals in Ophthalmic and Vision Research’.

### Antioxidant Preparation

#### I.P Injections

DCTEIO (20 mg/kg) was prepared by dissolution in absolute ethanol and then diluted 1:1 in injectable saline (50% final ethanol concentration). Vehicle injections consisted of 50% ethanol only. I.P injections (50 μL) were administered one hour prior and one hour post sham I/R or acute I/R insult.

#### I.O Injections

An initial 10 mM DCTEIO stock solution was prepared in DMSO before diluting to a 2.5 mM working solution in injectable saline. Assuming a vitreous volume of 20 μL [21], 2 μL was injected intraocularly to achieve a final DCTEIO concentration of 250 µM in the eye. I.O. injections were administered 30 min into the reperfusion phase. Equivalent volumes (2 μL) and concentrations (2.5% final DMSO) were administered to vehicle treated animals.

### Acute Retinal Ischaemia by Elevation of Intraocular Pressure

Unilateral ischaemia was induced as previously described [22, 23]. Briefly, after anaesthesia with 66 mg/kg ketamine (Ceva Animal Health, NSW, Australia) and 6.6 mg/kg xylazine (Ilium, Troy Laboratories, NSW, Australia) (I.P.), corneas were anaesthetised (0.4% oxybuprocaine hydrochloride, Minims, Bausch & Lomb, NSW, Australia) and the animal immobilized by resting the front teeth over a horizontal stabilizing bar and securing the skull with adjustable rods inserted in the bony ear canals. The anterior chamber was cannulated with a 30-gauge needle attached to a reservoir containing 0.9% NaCl. A micromanipulator was used to insert the needle into the anterior chamber parallel to the iris plane at the 12 o’clock position. Intraocular pressure (IOP) was increased to 120 mmHg by elevation of the reservoir to 163 cm. Ocular ischaemia was confirmed by the blanching of the iris and interruption of the retinal circulation. Corneal hydration was maintained throughout with GenTeal gel (Novartis, Alcon Laboratories, NSW, Australia). After 60 min of elevated IOP, removal of the cannula allowed reperfusion, generating oxygen radicals upon restoration of blood flow.

### Retinal Function – Electroretinography (ERG)

To investigate the capacity of DCTEIO to protect retinal function from the damaging effects of I/R injury, full-field ERGs were recorded as previously described [23].

ERGs were recorded on day 0 (pre-treatment) and day 8 (post-treatment). Briefly, rats were dark adapted overnight and prepared for recording under dim red light using LED illumination (λ_max_ = 650 nm). After anaesthesia (as above), pupils were dilated with 1% tropicamide and 2.5% phenylpherine (Minims, Bausch & Lomb). Reference electrodes were placed on each ear prior to positioning a platinum wire recording electrode on each cornea, which were kept moist with GenTeal gel. Body temperature was maintained at 37 °C with an electric animal heating blanket. Rats were then placed in a custom-designed ganzfeld and subjected to ERG flash stimuli. ERGs were recorded simultaneously from both eyes.

**Fig. 2:**
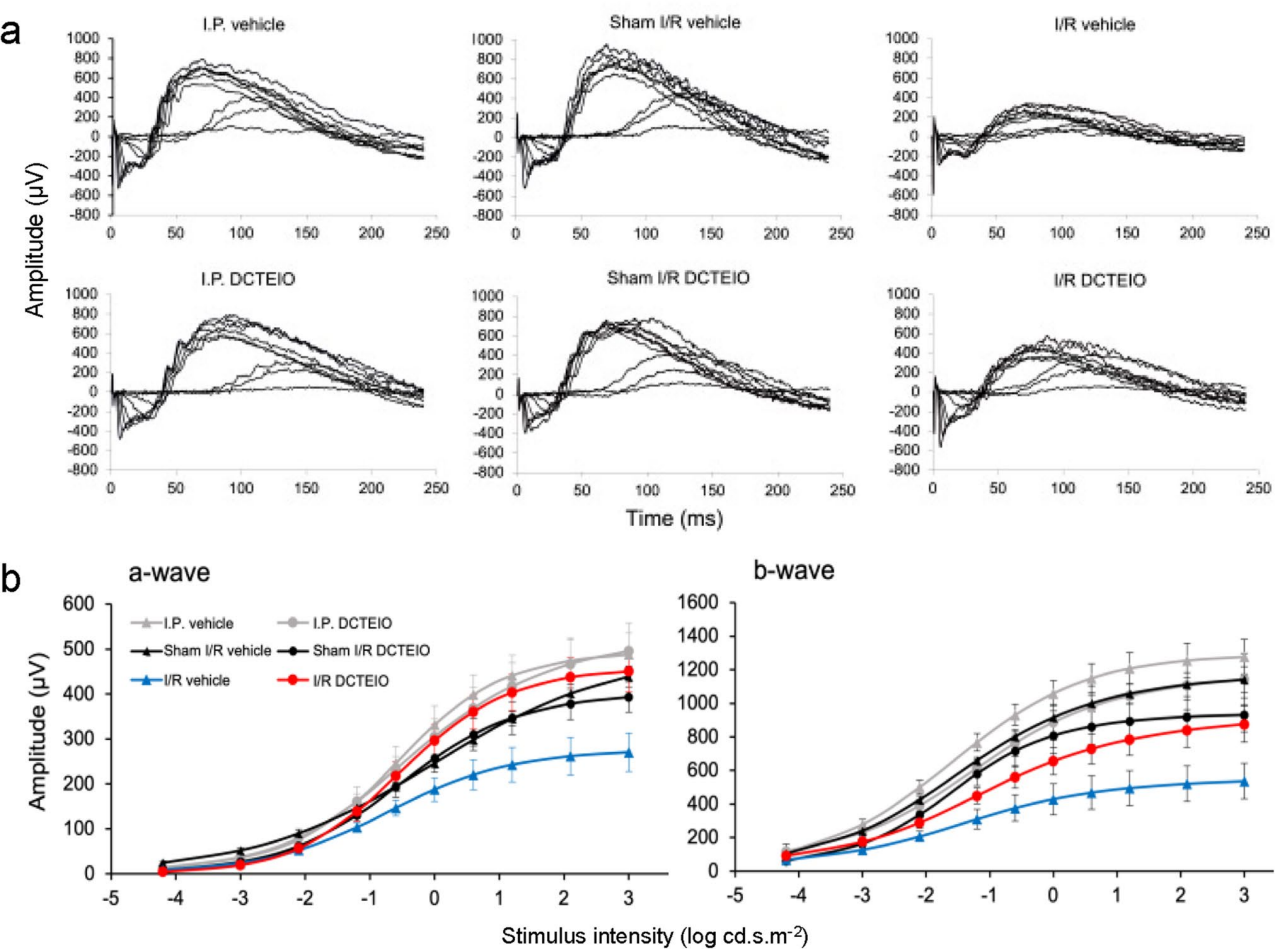
**a** Representative ERG waveforms elicited by flash stimulus intensities delivered over the range of -4.2 to 3.0 log cd.s.m^−2^ obtained from the control and experimental groups. **b** Mean ± SEM (n = 6) ERG amplitudes plotted as a function of increasing flash intensity and fitted with the Naka-Rushton equation to determine a- and b-wave *Rmax* values respectively

Flash stimuli were elicited with a photographic flash unit (Metz mecablitz 60CT4, Zirndorf, Germany) over a stimulus intensity range of − 4.2 to 3.0 log cd.s.m^−2^ (Fig. 2a), with sufficient interstimulus intervals (ranging from 10 to 360 s) to allow complete recovery of b-wave amplitudes. Responses were amplified and recorded with a bioamplifier/analogue-to-digital converter (Powerlab/4ST, AD Instruments, Castle Hill, Australia), band-pass filtered between 0.3 and 1000 Hz, and digitized at 4 kHz. The a-wave amplitude was measured from the baseline to the trough of the a-wave response and the b-wave amplitude was measured from the trough of the a-wave to the peak of the b-wave. Data were expressed as the mean wave amplitude ± SEM (µV). The mixed rod and cone a- and b-wave data were fitted with a Naka-Rushton equation [*R/R*_max_ = *I*^*n*^* / *(*I*^*n*^ + *K*^*n*^)] [24] (Fig. 2b) to determine *R*_max_ (maximum amplitude) from the response amplitude (*R*) and the flash intensity (*I*). A twin-flash paradigm was used to elicit and isolate cone-derived responses at 2.1 log cd.s.m^−2^_._ Oscillatory potentials (OPs) were isolated from a single flash (1.2 log cd.s.m^−2^) by digitally filtering the signal between 60 and 200 Hz. The maximum OP amplitude was calculated from the trough to the peak of the third OP (OP3).

Data presented (mean ± SEM, n = 6 per treatment group) represent the *R*_max_ amplitude of both a- and b-waves, cone responses and the maximum OP amplitude, for each treatment group.

### Immunohistochemistry

On day 8, animals were euthanized immediately following ERG analysis with > 200 mg/kg I.V Lethabarb (Virbac, NSW, Australia). Eyes were enucleated and fixed in 10% neutral buffered formalin (Sigma-Aldrich, NSW, Australia) for 2 h at room temperature. Posterior eye cups were halved through the optic nerve and one half cryoprotected with 30% sucrose (≥ 24 h) before mounting and freezing in OCT (Tissue Tek, ProSciTech, QLD, Australia) medium; the other half was prepared for histology (see below). Transverse sections (10 µm) were cut using a cryostat and maintained at − 20 °C until required for immunostaining, using standard methods. Slides were incubated overnight at room temperature with polyclonal rabbit anti-Glial Fibrillary Acidic Protein (GFAP, 1:1000, DakoCytomation, Glostrup, Denmark), or polyclonal rabbit anti-Ionized Calcium Binding Adaptor molecule 1 (IBA-1; 1:2000, Wako, Osaka, Japan) antibodies. Immunolabelling was visualised with Alexa Fluor 488 labelled goat-anti-rabbit IgG (1:300, Invitrogen, VIC, Australia), incubated for 90 min at room temperature. Images were viewed with a Nikon Eclipse Ti microscope (Tokyo, Japan) equipped with epifluorescence and captured with a Nikon DS-Qi2 camera. For GFAP quantification, photographs obtained approximately 2 mm superior to the optic disk were cropped to full retinal thickness and the mean grey value of each image was measured in Image J (I.P: vehicle control n = 4, sham I/R: DCTEIO n = 4, acute I/R injury: vehicle n = 6, acute I/R injury: DCTEIO n = 7). Only then were the representative images shown in Figs. 6 and 7 imported into Adobe Photoshop 22 for minor editing of contrast and sharpness.

As microglial activation is an early sign of neurodegenerative disease and inflammation that often precedes cell death, the distribution of IBA-1 positive microglia across the retina was quantified and classified into 3 segments: (1) inner retina (IR: consisting of the inner limiting membrane to the outer plexiform layer), (2) outer nuclear layer (ONL), and (3) photoreceptor inner and outer segments (PRS). Sample identities were masked and the microglia were counted by hand and categorised as either ‘ramified’, with small cell bodies and fine cellular processes, or ‘activated’, with larger cell bodies and short thicker processes, i.e. exhibiting an amoeboid-like morphology. Data are represented as the mean ± SEM (n = 3 for I.P & acute I/R, and n = 4 for I.O treatment groups).

### Histology

Fixed retinal samples were transferred to embedding cassettes, dehydrated with a graded ethanol series (70%, 90%, 100%) and xylene, and infiltrated with paraffin wax before embedding and cutting 3 µm sections. Slides were dried at 60 °C for 2 h, then allowed to cool at room temperature. Prior to staining, slides were deparaffinised and rehydrated (xylene, graded ethanol series: 100%, 90%, 70%, water). Slides were stained with Mayer’s haematoxylin (2 min) and blued in Scott’s water substitute solution (30 s), dehydrated and mounted. Images were viewed with a Nikon Eclipse Ti microscope (Tokyo, Japan) and captured with a Nikon DS-Fi2 camera. A minimum of 5 retinas from each treatment group were examined, with ≥ 3 images captured along the length of each retinal section. Similar retinal locations were imaged for each treatment group for comparison purposes.

### Cell Culture

The 661W photoreceptor cell line was kindly provided by Dr. Muayyad Al-Ubaidi (University of Oklahoma, AK, USA) and obtained from Dr. Krisztina Valter-Kocsi (Australian National University, ACT, Australia). Cells were initially established in T75 tissue culture flasks in Dulbecco Modified-Eagles medium (DMEM, Cat #: 10313-021, Gibco, Life Technologies, VIC, Australia) supplemented with 10% fetal bovine serum (FBS, Cat #: SH3084.03, Hyclone, Thermo Scientific, VIC, Australia), 2 mM L-glutamine (Cat #: 25030-081, Gibco, Life Technologies, VIC, Australia) and 50 U/ml penicillin, 50 µg/mL streptomycin (Cat #: 15070-063, Invitrogen, Life Technologies). Cells were cultured in a 37 °C incubator (5% CO_2_) and were harvested for experimental use upon reaching 80–85% confluence.

For experimental purposes, cells were seeded into T25 tissue culture flasks (1 × 10^5^ cells/flask) and allowed to adhere and proliferate in 10% FBS supplemented media for 24 h, before reducing the serum concentration to 1% FBS. This reduction in serum concentration slows the proliferation rate of the cells and promotes normal functionality, allowing the true effects of anti- and pro-oxidant treatments to be evaluated. Experiments were conducted on day 3 post seeding.

### ME-TRN Administration

The ME-TRN oxidative stress probe (100 nM) was prepared in 1% FBS-supplemented media (0.025% DMSO) before adding 2 mL to each T25 tissue culture flask. Cells were treated for 45 min to allow probe uptake and accumulation within the cells (Fig. 10a).

### Antioxidant Treatment

Cells were exposed to the established antioxidant lutein, and to the novel nitroxide-based antioxidant DCTEIO. The compounds were initially dissolved in DMSO and diluted in 1% FBS-supplemented media to achieve a range of experimental concentrations (0.1, 0.5, 1.0, 2.5, 5.0, 10, 20, 50, 100, 200 and 500 µM; 1.14% final DMSO concentration). To investigate the effect of the antioxidants, cells were treated with either vehicle (1.14% DMSO) or antioxidant, 30 min prior to inducing oxidative stress.

### Inducing Oxidative Stress with Antimycin (AMC)

Oxidative stress was induced in cell cultures by the cellular respiration (complex III) inhibitor antimycin (AMC). The ideal AMC concentration of 1 µM was established based on preliminary dose response studies (Fig. 10b). Cells were exposed to AMC and co-treated with the antioxidants for 15 min before analysis by flow cytometry.

### Flow Cytometry

Changes in ME-TRN fluorescence intensity in response to oxidative stress and antioxidant treatment were assessed by flow cytometry (MACSQuant Analyser 10, Miltenyi Biotec, NSW, Australia). Following treatment, cells were washed briefly in Hanks balanced salt solution (HBSS, Cat #: 14025134, Gibco, Life Technologies) and harvested from flasks using Versene (Cat #: 15040066, Gibco, Life Technologies) and TrypLE (Cat #: 12563011, Gibco, Life Technologies). Cell pellets were resuspended at a density of 1 × 10^6^ cells/mL in a Dulbecco’s phosphate-buffer saline (DPBS, 14040182, Gibco, Life Technologies) supplemented with 0.1% Bovine serum albumin (BSA, Cat #: A7030, Sigma-Aldrich, NSW, Australia) and 2 mM Ethylenediaminetetraacetic acid (EDTA, Cat #: E6758, Sigma-Aldrich). This solution aids cell survival and prevents cell clumping during flow cytometry.

Prior to running samples, MACSQuant calibration beads (Cat #: 130093607 MACSQuant, Miltenyl Biotec) were utilised to compensate for channel cross-talk. Voltages and scales were set for channel lasers and gating of desired cell populations (viable, single cells) determined utilising untreated control cells. Using the R-phycoerythrin (PE) channel, the mean fluorescent intensity (MFI) was determined for each sample based on 50,000 events, with an event referring to a cell falling within a set gate.

MFIs were initially expressed as a percent (%) fluorescence based upon the ME-TRN control. Data shown (mean ± SEM, minimum of 5 replicate experiments) represent the % mitigation offered by antioxidant treatment based upon the % reduction in fluorescence of AMC treated cells alone (Fig. 10d).

### Data Analysis

GraphPad Prism 9 software was used for statistical analysis and figure creation. ERG data represent the *R*_*max*_ amplitude of the mixed (rod and cone) a- and b-waves, the isolated cone b-wave response and maximum OP amplitude, expressed as the mean ± SEM (n = 6). For IBA-1 cell counting, each group consisted of at least 3 animals with images counted in duplicate. Microglia were categorized as being either ‘ramified’ or ‘active’ and present in either the IR, ONL or PRS. Data (mean + SEM) are expressed as either ‘total cell counts’ across the entire retina, or as ‘ramified and active’ across either the entire retina, or within the defined IR, ONL and PRS. Flow cytometry data (mean ± SEM, n ≥ 5), represents the % mitigation offered by antioxidant treatment against AMC treated cells alone.

**Fig. 3:**
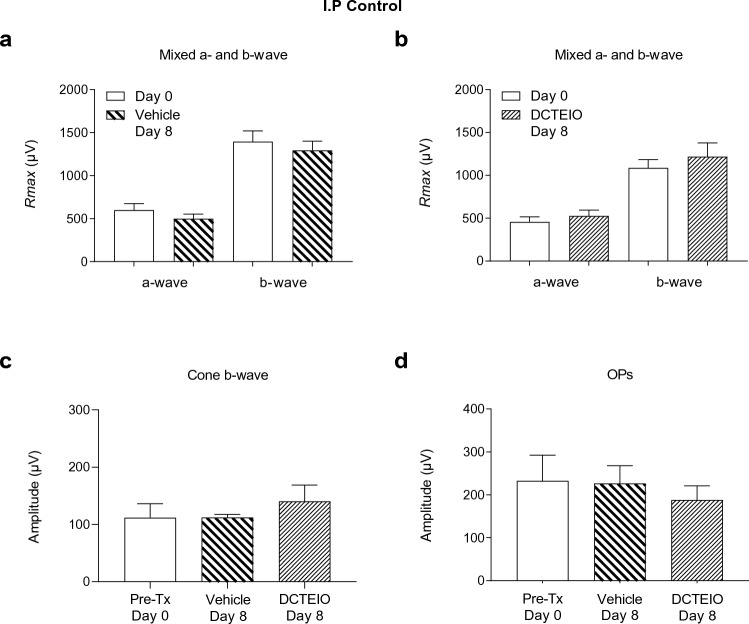
ERG analysis of vehicle (50% ethanol) and DCTEIO (20 mg/kg) treated animals administered I.P injections alone, 0 (pre-treatment) and 8 days post-treatment (mean ± SEM, n = 6). Graphs represent the *R*_*max*_ amplitude for a- and b-waves (**a** & **b**), cone-isolated b-wave (2.1 log cd.s.m^−2^) (**c**) and oscillatory potentials (OPs) (1.2 log cd.s.m^−2^) (**d**) responses. These data confirm no significant systemic effect of vehicle or DCTEIO administration on normal retinal function

**Fig. 4:**
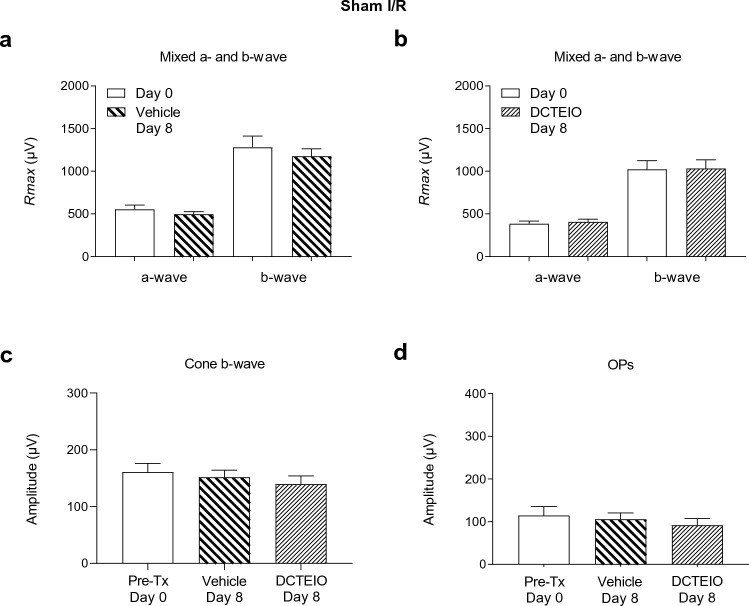
ERG analysis of vehicle (50% ethanol I.P; 2.5% DMSO I.O) and DCTEIO (20 mg/kg I.P; 250 µM I.O) treated animals subjected to ‘sham I/R’ injury, 0 (pre-treatment) and 8 days post-treatment (mean ± SEM, n = 6). Graphs represent the *R*_max_ amplitude for a- and b-waves (**a** & **b**), cone-isolated b-wave (2.1 log cd.s.m^−2^) (**c**) and oscillatory potentials (OPs) (1.2 log cd.s.m^−2^) (**d**) responses. These data confirm that an I.O injection of vehicle or DCTEIO has no effect on normal retinal function

**Fig. 5:**
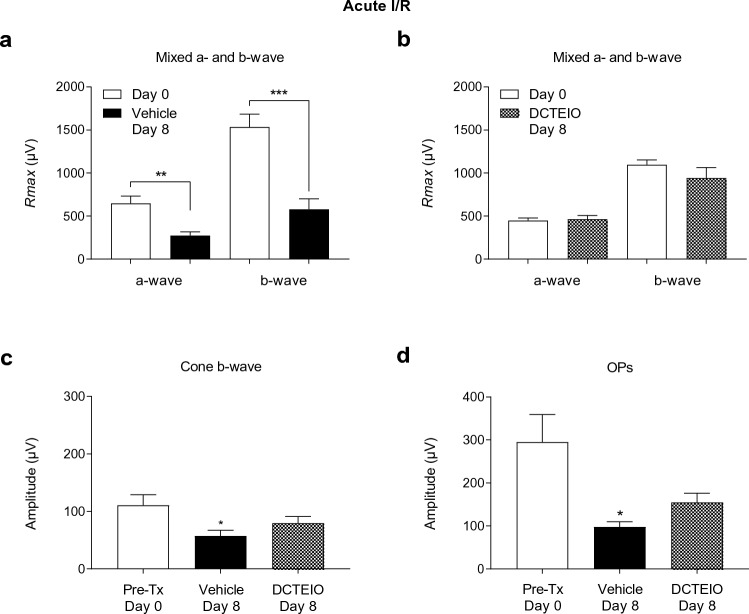
ERG analysis of vehicle (50% ethanol I.P; 2.5% DMSO I.O) and DCTEIO (20 mg/kg I.P; 250 µM I.O) treated animals, 0 (pre-treatment) and 8 days post I/R injury (mean ± SEM, n = 6). Graphs represent the *R*_max_ amplitude for a- and b-waves (**a** & **b**), cone-isolated b-wave (2.1 log cd.s.m^−2^) (**c**) and oscillatory potentials (OPs) (1.2 log cd.s.m^−2^) (**d**) responses. A significant decrease in a- and b-wave amplitude was observed in ‘acute I/R injury:vehicle’ treated animals 8 days post-treatment (**a**), when compared to pre-treatment, day 0 (**p = 0.0028, ***p = 0.0006) data. Administration of the antioxidant DCTEIO blunted the damaging effects of I/R on retinal function (**b**), with no significant differences detected between all treatment groups. DCTEIO partially protected the cone-isolated b-wave (**c**) and OP (**d**) responses from I/R insult, with post-treatment response amplitudes no longer significantly different from the pre-treatment control data (cones: *p* = 0.2807, OPs: *p* = 0.06)

Data were tested for normality with the Shapiro–Wilk test. Statistical comparisons were made using an unpaired t-test for Figs. 3, 4 and 5 images a & b, and an ordinary one-way ANOVA, with Sidak’s (ERG & IBA-1) or Dunnett’s (flow cytometry) multiple comparisons test. *P* values less than 0.05 were considered statistically significant.

## Results

### Preservation of Retinal Function: Electroretinography

Control rats administered vehicle or DCTEIO by I.P injection alone, were initially investigated to determine any possible significant systemic effects of drug administration on retinal function. Figure 3 demonstrates no significant difference in a- or b-wave amplitudes between day 0 and day 8 recordings, in both vehicle (Fig. 3a: a-wave: 599 ± 77 µV vs 500 ± 52 µV; *p* = 0.3105, b-wave: 1397 ± 124 µV vs 1294 ± 107 µV; *p* = 0.5421) and DCTEIO (Fig. 3b: a-wave: 457 ± 59 µV vs 527 ± 68 µV; *p* = 0.4590, b-wave: 1086 ± 98 µV vs 1219 ± 158 µV; *p* = 0.4892) treatment groups. Similarly, cone and oscillatory potential responses were not affected by the I.P administration of vehicle or DCTEIO (Fig. 3c: cones: *p* = 0.5872, Fig. 3d: OPs: *p* = 0.7629).

Figure 4 shows that the addition of an I.O injection of vehicle or DCTEIO to ‘sham I/R’ animals had neither a positive nor negative effect on a- or b-wave (Fig. 4a: vehicle: a-wave: 555 ± 50 µV vs 496 ± 31 µV, *p* = 0.3423; b-wave: 1281 ± 131 µV vs 1176 ± 85 µV, *p* = 0.5163; Fig. 4b: DCTEIO: a-wave: 386 ± 30 µV vs 406 ± 32 µV; *p* = 0.6606, b-wave: 1023 ± 101 µV vs 1032 ± 102 µV; *p* = 0.9467), cone (Fig. 4c: *p* = 0.5915) and OP (Fig. 4d: *p* = 0.6587) responses, compared with pre-treatment values.

Vehicle treated rats subjected to ‘acute I/R’ injury (Fig. 5) displayed signs of retinal dysfunction with significant decreases observed in both a-wave and mixed (rod and cone) b-wave amplitudes at 8 days post treatment (Fig. 5a: a-wave: 647 ± 86 µV vs 275 ± 40 µV; **p = 0.0028, b-wave: 1534 ± 150 µV vs 580 ± 122 µV; ***p = 0.0006, for day 0 and day 8, respectively). DCTEIO inhibited the ROS-induced suppression of both the a-wave and mixed (rod and cone) b-wave that was observed in vehicle treated animals (Fig. 5b: a-wave: 449 ± 29 µV vs 463 ± 43 µV; *p* = 0.7894, b-wave: 1095 ± 58 µV vs 942 ± 122 µV; *p* = 0.2815, for day 0 and day 8 data, respectively). Similarly, DCTEIO administration partially protected the cone-isolated b-wave response from the I/R insult, such that the post-treatment response amplitude (80 ± 12 µV) was no longer significantly different from the non-ischaemic pre-treatment control (111 ± 18 µV; *p* = 0.2807, Fig. 5c). Figure 5d shows that I/R injury significantly reduced the mean OP amplitude from 295 ± 64 µV (day 0 pre-treatment) to 98 ± 12 µV (day 8 post-treatment). Whilst the apparent increase in OP amplitude to 155 ± 21 µV with DCTEIO administration was not statistically significantly different from responses recorded from the ‘acute I/R: vehicle’ treated animals, it was also not significantly different from the pre-treatment day 0 OP amplitudes (*p* = 0.06, Fig. 5d).

### Immunohistochemistry and Cell Counts

**Fig. 6:**
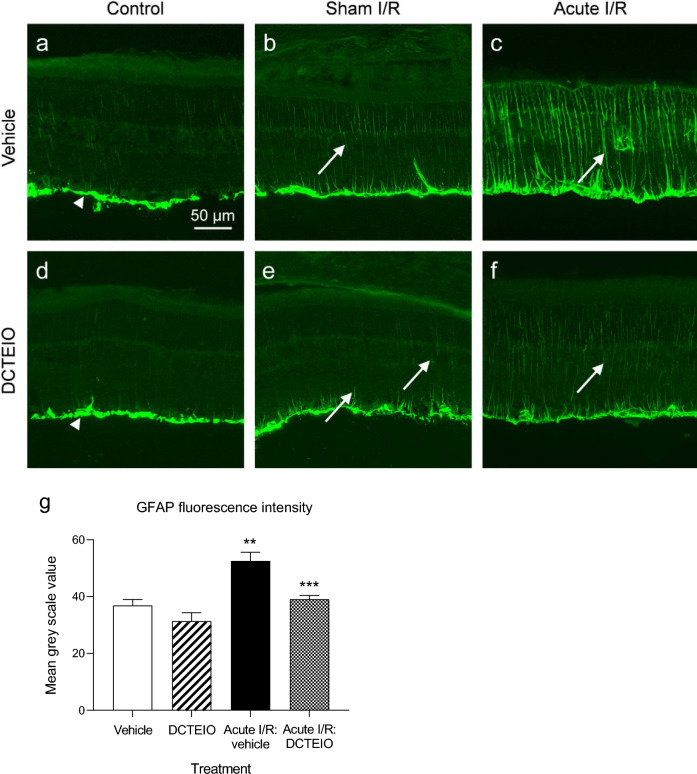
GFAP immunohistochemistry (**a**–**f**) of rat retinas with fluorescence quantification (**g**), 8 days post-treatment. No differences in labelling were apparent between ‘I.P: vehicle’ (**a**) and ‘I.P: DCTEIO’ (**d**) animals, with GFAP expression predominantly restricted to the astrocytes and Müller cell endfeet in the ganglion cell/nerve fibre layer (arrowheads). An intraocular injection in ‘sham I/R’ animals, resulted in minor activation of the Müller cells (**b**, **e** and **g**, arrows). In contrast, an ischaemic insult (acute I/R: vehicle) followed by 8 days of reperfusion resulted in a significant upregulation in GFAP immunoreactivity associated with activated Müller cells (**c** and **g**, **p = 0.0011, n = 6, arrows). The administration of DCTEIO substantially reduced GFAP upregulation with only minor activation of the Müller cells (**f** and **g**, ***p = 0.0004 compared with acute I/R: vehicle, n = 6)

To investigate retinal glial cell activation induced by acute I/R injury and the protection offered by DCTEIO, glial cell activation was analysed as a hallmark of reactive gliosis and neuroinflammation by GFAP (macroglia) and IBA-1 (microglia/macrophage) immunostaining. Figure 6 shows that GFAP expression in I.P: vehicle and I.P: DCTEIO treated ‘control’ animals was predominantly restricted to astrocytes and Müller cell endfeet in the ganglion cell/nerve fibre layer (Fig. 6a, d). This expression is identical to that seen in ‘normal’ untreated eyes (not shown). The intraocular injection of vehicle or DCTEIO (250 µM) to the ‘sham I/R’ treatment groups (Fig. 6b, e) did however induce a slight expression of GFAP in the Müller cell processes. In contrast, acute I/R injury resulted in a significant upregulation of GFAP expression in vehicle treated animals (Fig. 6c, g, **p = 0.0011) at 8 days post treatment. Figure 6c shows the GFAP distribution in glial processes from the inner limiting membrane to the outer retina indicative of activated gliotic Müller cells. This upregulation of GFAP in the Müller cells was significantly reduced by treatment with the nitroxide antioxidant DCTEIO (Fig. 6f, g, ***p = 0.0004).

**Fig. 7:**
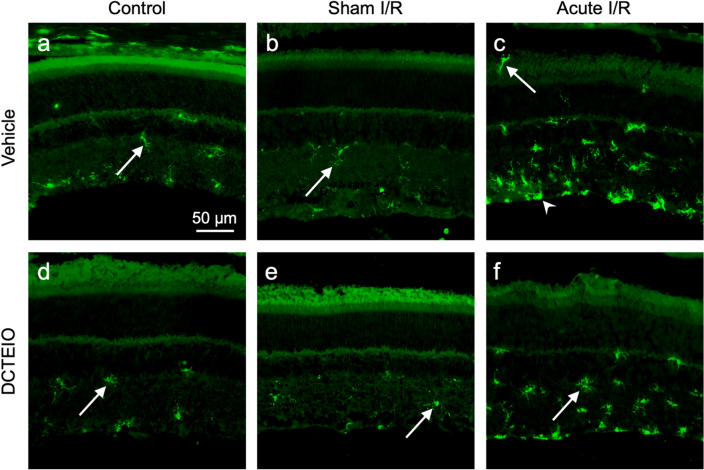
IBA-1 immunohistochemistry of rat retinas 8 days post-treatment. ‘I.P’ (**a** & **d**) and ‘sham I/R’ treated animals (**b** & **e**) displayed limited numbers of microglia in a ‘resting ramified’ state (white arrows). The induction of ischaemia (acute I/R) resulted in an upregulation of IBA-1 expression, increased microglial migration and numerous microglia displaying a small, spherical, amoeboid-like morphology indicative of ‘reactive’ microglia (**c**, white arrowhead). DCTEIO successfully reduced the overall number of ‘reactive’ microglia (**f**), ultimately reducing the immunological response

**Fig. 8:**
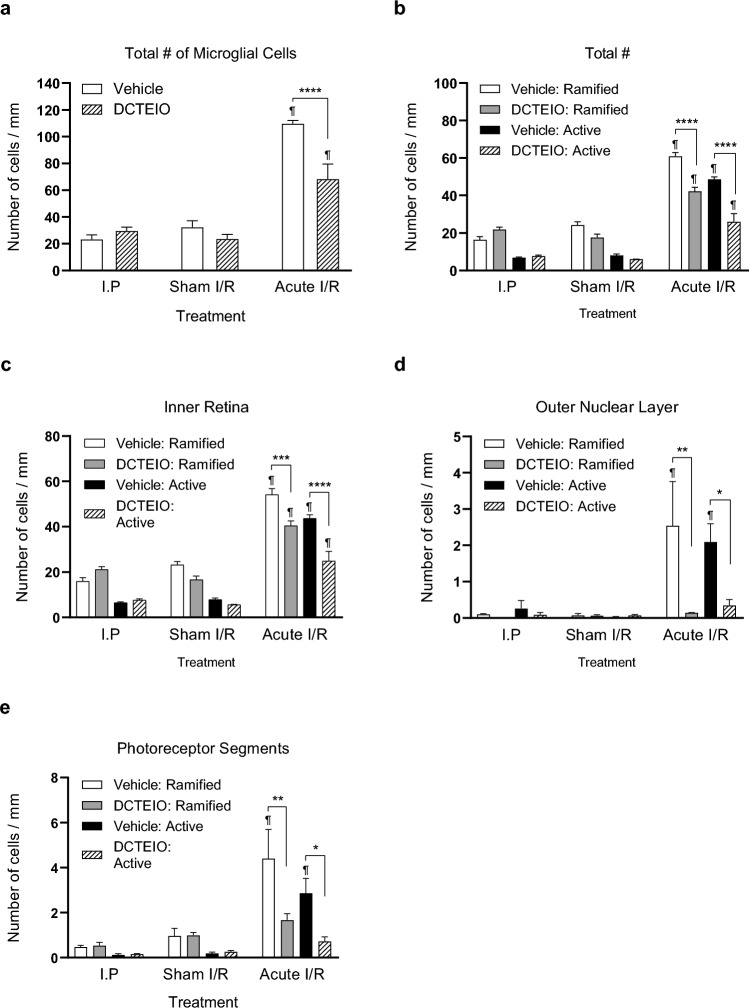
The effect of DCTEIO on the number and distribution of ‘ramified’ (resting) and ‘active’ (amoeboid) microglial cells in the retina 8 days post treatment (mean ± SEM, n = 3 for I.P & acute I/R, and n = 4 for I.O treatment groups). DCTEIO significantly reduced both classes of microglia in the inner retina (IR), outer nuclear layer (ONL) and photoreceptor segments (PRS) in ‘acute I/R treated animals. *p < 0.05, **p < 0.005, ***p < 0.0005, ****p < 0.0001. ^¶^*p* < 0.0001 when comparing ‘acute I/R’ and ‘sham I/R’ treatment groups

Figures 7 and 8 demonstrate the reactivity of the microglia in response to retinal injury. Here limited numbers of total microglia were observed in both ‘I.P’ (Fig. 7a, d) and ‘sham I/R’ treated animals (Fig. 7b, e) for both vehicle and DCTEIO treatment groups, when compared to those subjected to ‘acute I/R’ injury (Fig. 7c, f). Quantitative analysis of total microglial cell numbers (Fig. 8a) within ‘I.P’ and ‘sham I/R’ treatment groups showed no significant difference between vehicle and DCTEIO treated animals (I.P: 23.15 ± 1.97 vs 29.49 ± 1.64, *p* = 0.7433; sham I/R: 32.26 ± 2.46 vs 23.55 ± 1.68, *p* = 0.2505, for vehicle and DCTEIO respectively). Moreover, an I.O injection of either vehicle or DCTEIO did not induce an immunological response within the retina when compared to the ‘I.P’ treatment group (vehicle: *p* = 0.2822 and DCTEIO: *p* = 0.7412).

Figure 8a demonstrates that ‘acute I/R’ injury induced a significant upregulation in total microglial cell numbers when compared to ‘sham I/R’ treatment groups (109.6 ± 1.45 vs 32.26 ± 2.46, ^¶^*p* < 0.0001, for ‘acute I/R’ and ‘sham I/R’ respectively), and that treatment with DCTEIO significantly reduced the migration and/or accumulation of these cells following I/R injury (109.6 ± 1.45 vs 68.17 ± 6.56, ****p < 0.0001, for vehicle and DCTEIO respectively).

In addition to enhanced IBA-1 expression and influx of migratory microglia following I/R injury, Fig. 7c demonstrates that a stepwise de-ramification of the microglia occurred, with ramified microglia transforming into either an ‘activated state’ characterised by swollen cells with a larger cell body and shorter, thicker processes, or alternatively a ‘reactive state’ typically characterised as small, spherical cells exhibiting an amoeboid-like morphology (white arrowhead). Quantitatively, Fig. 8b shows that ‘acute I/R’ animals treated with DCTEIO not only significantly reduced the total number of infiltrating ramified microglia (61.00 ± 1.89 vs 42.22 ± 2.26, ****p < 0.0001, for vehicle and DCTEIO respectively), but also significantly reduced the total number of microglia becoming activated (48.59 ± 1.406 vs 25.95 ± 4.376, ****p < 0.0001 for vehicle and DCTEIO respectively), thus reducing the overall immunological response within the retina.

To determine the location in which this protection was achieved, ‘ramified’ and ‘active’ cell numbers were separated into being present in either the IR (Fig. 8c), ONL (Fig. 8d) or PRS (Fig. 8e). DCTEIO significantly reduced both the number of ramified (IR: 54.05 ± 2.76 vs 40.41 ± 2.12, ***p = 0.0004, ONL: 2.543 ± 1.22 vs 0.140 ± 0.02, **p = 0.0012 and PRS: 4.403 ± 1.30 vs 1.669 ± 0.28, **p = 0.0019 for vehicle and DCTEIO respectively) and active (IR: 43.64 ± 1.60 vs 24.89 ± 4.26, ****p < 0.0001, ONL: 2.089 ± 0.51 vs 0.345 ± 0.16, *p = 0.0387 and PRS: 2.863 ± 0.66 vs 0.720 ± 0.20, *p = 0.0267 for vehicle and DCTEIO respectively) microglia within each retinal segment following I/R injury, with ‘ramified’ and ‘active’ numbers no longer significantly different from ‘sham I/R’ microglial numbers in both the ONL (ramified: 0.140 ± 0.02 vs 0.064 ± 0.03, *p* > 0.9999 and active: 0.345 ± 0.16 vs 0.070 ± 0.03, *p* > 0.9999 for ‘acute I/R’ and ‘sham I/R’ respectively) and PRS (ramified: 1.669 ± 0.28 vs 0.988 ± 0.13, *p* > 0.9955 and active: 0.720 ± 0.20 vs 0.25 ± 0.06, *p* > 0.9999 for ‘acute I/R’ and ‘sham I/R’ respectively)*.* These data suggest that DCTEIO not only significantly reduced the number of microglia migrating through to the ONL and PRS, but also significantly reduced the number of microglia within those retinal segments becoming activated.

### Histology

**Fig. 9:**
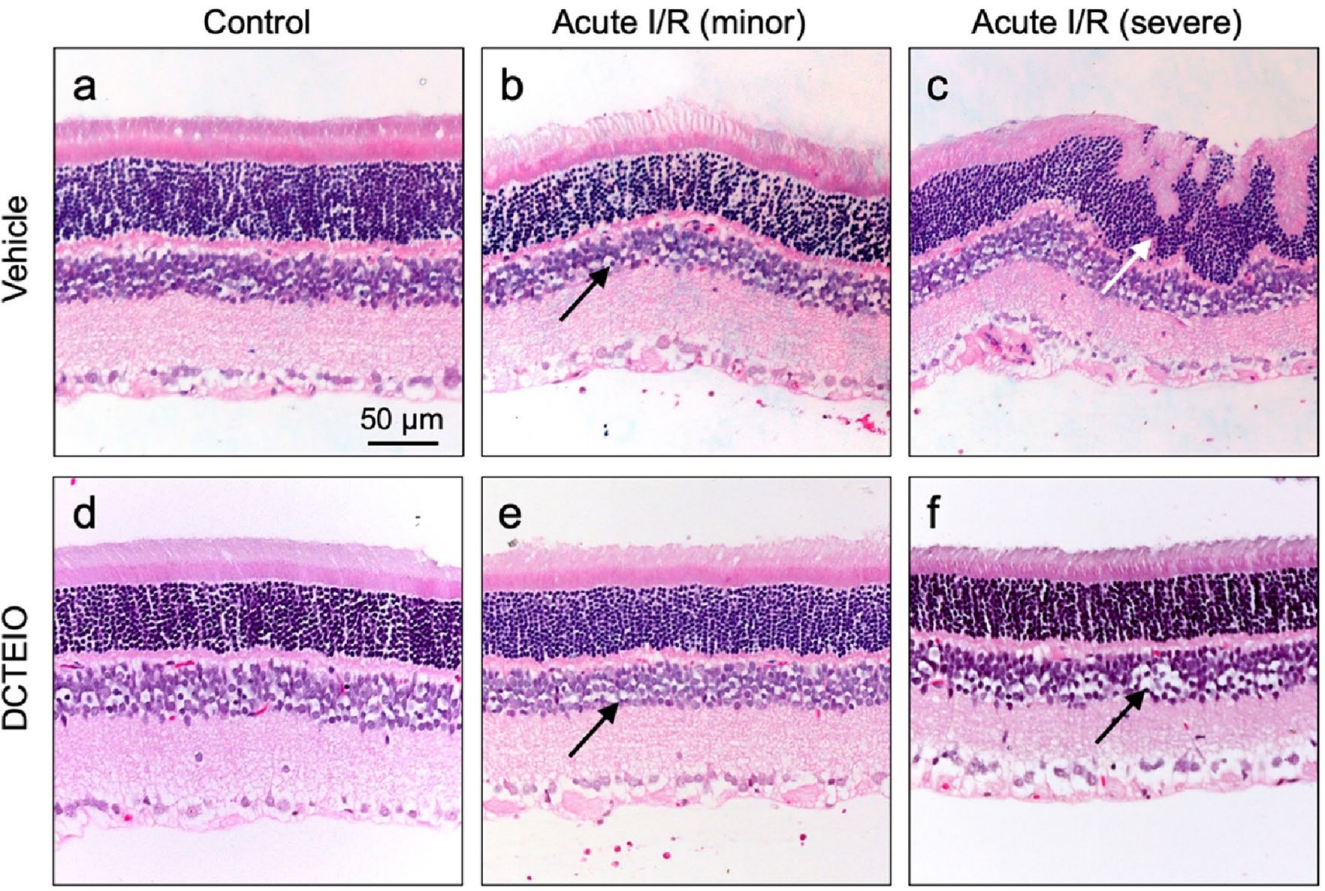
Hematoxylin–eosin staining of rat retinas 8 days post-treatment. Undamaged sham I/R: vehicle (**a**) and sham I/R: DCTEIO (**d**) retinas. Damage to ‘acute I/R: vehicle’ treated retinas varied from minor cell loss (**b**, black arrow), to complete disruption and disorganisation of the nuclear layers (**c**, white arrow). DCTEIO preserved the structural integrity of each cell layer, with minor to indiscernible cell loss observed in the inner nuclear layer (**e** and **f**, black arrows)

Examination of retinal sections confirmed that the extent of damage in ‘acute I/R: vehicle’ treated retinas and the protection offered by DCTEIO, varied along a continuum across the fundus within each eye. Figure 9 shows examples of the extremes of that continuum and demonstrates that the extent of damage to I/R treated retinas varied from minor edema/cell loss in both the inner and outer nuclear layers (Fig. 9b) to complete disruption and disorganisation of the nuclear layers, particularly to the outer segment, sometimes with the formation of retinal folds (Fig. 9c, white arrow). Although edema/cell loss was also observed in ‘acute I/R: DCTEIO’ treated retinas, it appeared to be restricted to the inner nuclear layer, with varying degrees of damage (Fig. 9f) to almost undetectable (Fig. 9e) quantities.

### Flow Cytometry: Quantification of Oxidative Stress

**Fig. 10:**
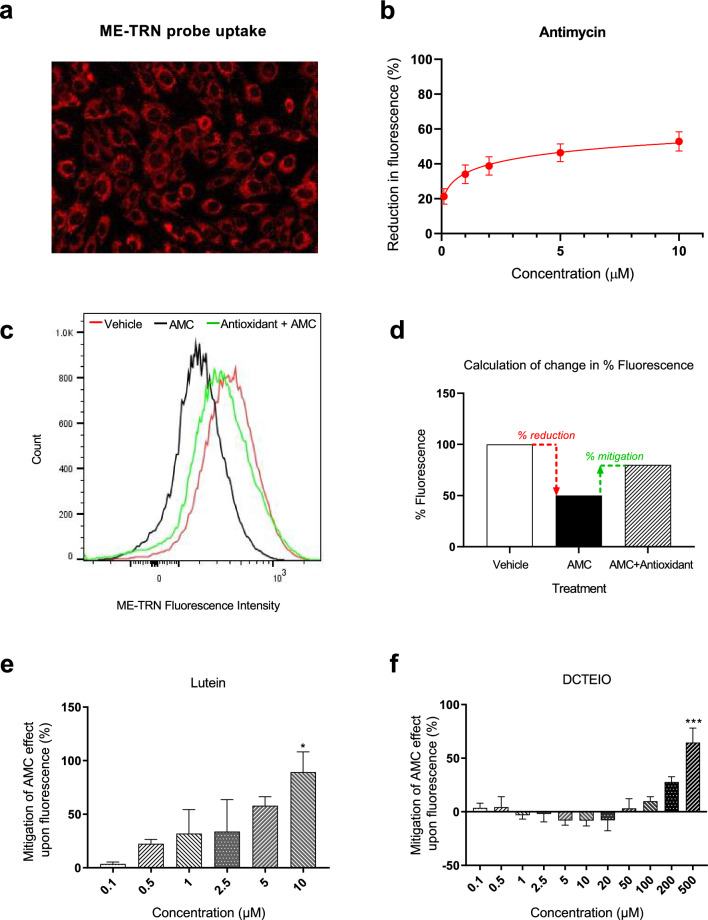
Quantification of oxidative stress and the protection offered by antioxidant compounds in the 661W photoreceptor cell line by flow cytometry. **a** Accumulation of red fluorescent ME-TRN probe by the mitochondria of cultured 661W cells. **b** Dose–response characteristics of the mitochondrial complex III inhibitor, antimycin (AMC), in the reduction of ME-TRN MFI. **c** Histograms of the MFI for vehicle (red), AMC 1 µM (black) and antioxidant (green) treated cells. **d** Example of calculating change in % fluorescence (% reduction and % mitigation). **e** Mitigation of the AMC-induced pro-oxidant effect upon ME-TRN fluorescence by the carotenoid antioxidant lutein and the nitroxide antioxidant DCTEIO (**f**). Data are expressed as the mean ± SEM (n ≥ 5). *p = 0.0143, ***p = 0.0002

It has been previously demonstrated that healthy retinas and cultured cells convert the ME-TRN probe from a non-fluorescent oxidised state to a reduced fluorescent state during normal metabolism [17, 18, 25]. Figure 10a shows the accumulation of the probe into the mitochondria of 661W photoreceptor cells. When subjected to oxidative stress, the probe is converted back to a more oxidised free radical state, resulting in a decrease in probe fluorescence [25]. Using this unique ability of the probe to interchange between states based on redox status, a rapid technique was developed using flow cytometry to quantify oxidative stress and the mitigation offered by antioxidant compounds in an in vitro model.

To determine the appropriate concentration of AMC with which to induce a significant oxidative stress but without a toxic effect on cell viability, 661W cells were challenged with AMC at a range of concentrations (0.1 to 10 μM) for 15 min. Figure 10b demonstrates the dose-dependent effect of AMC upon reduced ME-TRN probe fluorescence (MFI), with the maximum effect apparent at 10 µM. We determined an approximate IC_50_ of 1 µM AMC, which was subsequently used for all further experiments.

Lutein is a potent carotenoid antioxidant that can improve vision and retard the progression of AMD and cataract development. In vitro studies show that it can penetrate into cells and scavenge intracellular H_2_O_2,_ thereby preventing cell damage [26]. Accordingly, we incorporated the use of lutein as a positive antioxidant control. Figure 10e demonstrates the dose-dependent capacity of lutein to mitigate the pro-oxidant effect of AMC upon 661W cells, as quantified by the MFI of the ME-TRN-loaded cells when analysed by flow cytometry.

Figure 10f demonstrates that the nitroxide-based antioxidant DCTEIO ameliorated the pro-oxidant effect of AMC, significantly mitigating the suppressive effects of AMC upon ME-TRN probe fluorescence at 500 µM (Fig. 10f: 64.56 ± 13.47%, ***p = 0.0002).

## Discussion

It is essential that ROS production is offset by ROS removal for cellular health to be maintained [27]. The excessive generation of ROS by both endogenous (mitochondrial electron transport chain [28], NADPH oxidases [29, 30], cytochrome P450 oxygenases, xanthine oxidases, lipoxygenase and cyclooxygenase [28, 31] and/or exogenous (tobacco smoke, fatty acids, transition metals and ethanol) sources, causes neuronal damage and the subsequent release of cytosolic factors that activate neighbouring glial cells (microglia and astrocytes) [32]. These cells respond by releasing proinflammatory cytokines as well as ROS and RNS, further promoting the inflammatory response, exacerbating the neuronal damage, and ultimately creating an amplification loop leading to chronic neurodegeneration [32]. The search for new protective remedies has been focused on molecules combining antioxidant utilities with recycling capacities [33], with cyclic nitroxides fulfilling this requirement [34]. Nitroxides have been identified as novel antioxidants protecting isolated macromolecules, cells, organs and whole animals from diverse insults [35–40]. Here the ‘long-term’ protection offered by a novel nitroxide-based antioxidant DCTEIO in the rat retina in vivo was investigated*.*

Firstly, it was demonstrated that the administration of the nitroxide antioxidant DCTEIO (20 mg/kg) does not affect normal retinal function over a period of 8 days as evidenced by the stability of the ERG a- and b-wave, isolated-cone and OP amplitudes (Fig. 3). This suggests that DCTEIO is not toxic to the retina at this concentration. Ischaemic-reperfusion injury is known to induce long-term functional deficits to the retina, particularly a suppression of the ERG rod and cone b-waves and OPs [23, 41] in part due to the generation of excessive ROS during both the acute ischaemic phase [42] and the reperfusion phase [43]. This present study demonstrates that DCTEIO significantly reduces the negative impacts of I/R injury and maintains retinal function at normal, pre-injury levels, for at least 8 days after an I/R injury (Fig. 5). Consistent with the observed preservation of retinal function, histological analysis also showed preservation of retinal structural integrity, particularly that of the inner and outer nuclear layers, from where these ERG a- and b-wave responses are respectively derived (Fig. 9).

In the mammalian retina, the glial Müller cells only express very low levels of GFAP (Fig. 6a) [44]. The upregulation of GFAP is an extensively reported marker for reactive Müller cells and as an indicator of retinal stress, retinal injury and Müller cell activation. Acute I/R injury results in a significant upregulation of GFAP expression, enhancing GFAP distribution to Müller cell processes extending from the inner limiting membrane to the outer retina (Fig. 6c). This response of the Müller cells is significantly blunted by DCTEIO (Fig. 6f), providing further evidence of its protective capacity against I/R induced retinal damage.

In chronic neurodegenerative diseases including those of the retina, microglial activation is an early sign that often precedes neuronal death. Accordingly, the targeting of microglia for the treatment of retinal degenerative diseases has been proposed [45]*.* In response to cellular stressors including oxidative stress [46], microglia become activated, progressing from a surveillance ‘resting’ mode to an activated effector phenotype [47]. Morphological change is often accompanied by migration and proliferation, with microglial cells invading nuclear layers (particularly the ONL) commonly devoid of immune cells [47–51]. Here similar effects were observed and quantified, including the appearance of microglia in the outer retina following I/R injury (Figs. 7, 8). Although DCTEIO did not totally mitigate the proliferation and/or infiltration and migration of microglial cells to the retina, it significantly reduced the overall number of microglia transforming to an ‘activated/reactive state’. This effect of DCTEIO upon glial cell activation is significant in two ways due to the interdependent cell signalling pathways in the retina. Firstly, reduced microglial activation is suggestive of fewer dying cells, thereby a reduced requirement for phagocytosis of cellular debris. Secondly, reduced microglia activation per se would probably reduce the further secretion of proinflammatory cytokines including IL-1ß, likely leading to reduced inflammasome activation and consequent cell death. Furthermore, the reduction of I/R-induced Müller cell activation, as evidenced by lower GFAP expression following DCTEIO administration (Fig. 6f, g), likely alters microglial activity and vice-versa, due to the cellular crosstalk between retinal glia [45]. Together, these data suggest multiple potential cellular targets for the neuroprotective actions of DCTEIO.

The protection offered by DCTEIO is mediated by several of the advantageous properties attributed to nitroxide antioxidants: other nitroxides are in development as preventative or therapeutic pharmaceutical drugs for a variety of neurodegenerative diseases [6]. The protective effect of nitroxides has been elucidated in experiments beginning in the 1980’s [11] and shown to be the result of their broad antioxidant activity both in vitro and in vivo [52–55]. More recently, nitroxides have also been found to possess anti-inflammatory and anti-angiogenic properties, strengthening their potential use as effective drugs against oxidative stress related pathologies [52, 56], including the common retinal diseases e.g. AMD and diabetic retinopathy, whose aetiologies include oxidative stress, inflammation and angiogenesis. Nitroxides have a low molecular weight, are non-toxic, do not elicit immunogenic effects on cells [57], and when devoid of electrical charge can easily diffuse through cell membranes allowing the direct targeting of ROS formed within the cells [56, 58]. Nitroxides also accept electrons from mitochondrial electron transport-chain proteins, thereby inhibiting pathological ROS accumulation [14]. The antioxidant action of nitroxides is therefore associated with regulation of the redox state within the cell [54, 56].

In biological conditions, nitroxides mimic superoxide dismutase (SOD), modulate hemoprotein’s catalase-like activity, scavenge reactive free radicals, inhibit the Fenton and Haber–Weiss reactions, and supress the oxidation of biological materials such as peptides, protein, and lipids [14, 15, 52, 54, 56]. Nitroxides, unlike other antioxidants, are characterised by a catalytic mechanism of action associated with a reversible, single electron oxidation and reduction reaction [56, 59]. DCTEIO may be considered a derivative of CTMIO [18, 60] modified using larger ethyl substituents over more typical (and smaller) methyl groups present in nitroxides such as CTMIO or TEMPOL. The larger ethyl groups mean that DCTEIO is reduced at a slower rate intracellularly than tetramethyl analogues. The larger ethyl groups surrounding the nitroxide radical help to physically shield the redox-active -NO component of the nitroxide from reaction with cellular reductants. Slowing down the rate of reduction serves to deliver a higher effective and longer lasting concentration of the radical under conditions of oxidative stress. These factors can have significant effects on their rate of reduction and the ability of a nitroxide to scavenge and dismutate ROS [61].

Nitroxides with anionic substituents are more resistant to reduction than neutral compounds which are in turn more stable than cationic substituted nitroxides [62]. In that context, nitroxides with carboxylic acid substituents (e.g. DCTEIO) typically have one of the slowest reduction rates in vivo [61]. However compounds adorned with charged substituents in general (e.g. carboxylic acid and amine salts) are predominately water soluble and consequently have limited ability to pass thorough phospholipid membranes [63]. Uptake is possible via the non-dissociated uncharged form (RCOOH), a small amount of which is always present in equilibrium with the dissociated anionic salt, depending on the pH of the environment.

We confirmed that the neuroprotective effects of DCTEIO observed in vivo could be ascribed to both its known antioxidant capacity in vivo [18] and also in cultured retinal cells (Fig. 10). A rapid in vitro technique was developed using a redox-responsive, nitroxide-based probe (ME-TRN) and flow cytometry to quantify oxidative stress in cultured photoreceptor cells. ME-TRN is rapidly and selectively accumulated by the mitochondria, the cells’ main energy source and hence largest producers of ROS. The ME-TRN probe differs from other commercially available ROS dyes, e.g. CellROX (ThermoFisher), in that it is reversibly-responsive to both pro- and anti-oxidant stimuli [17, 18, 25]. Various cellular redox processes can mediate the conversion between the oxidized nitroxide species and reduced hydroxylamine, and hence the ratio of the two states is indicative of the overall ‘reducing capacity’, or redox environment, of the cell [17, 64–67]. Figure 10e and f suggest that a high dose of DCTEIO (compared to lutein) is needed to mitigate the oxidative stress induced by AMC. Presumably, a higher concentration of DCTEIO is necessary to overcome its reduced lipophilicity and ability to permeate membranes in vitro which in turn impacts on its capacity to interact with crucial ROS in the cell. Furthermore, the decreased reduction rates of the nitroxide arises from the size of the tetraethyl substituents, however this increased size may also restrict access to the NO functionality that imparts ROS scavenging capacity therefore requiring higher concentration for effective reaction rates. Notably, although reduction is expected to be slower than for less hindered tetramethyl analogues, the more structurally restricted nitroxide DCTEIO can still shuttle between the reduced and oxidised states thus allowing catalytic turnover which continuously restores the nitroxide as a functioning ROS scavenger over many cycles. These factors are expected to explain why DCTEIO has long-term in vivo effects at these concentrations.

## Data Availability

The datasets generated and/or analysed during the current study are not publicly available but are available from the corresponding author on reasonable request.
